# Differences in Chronic Pulmonary Embolism Burden in Chronic Thromboembolic Disease with and Without Pulmonary Hypertension

**DOI:** 10.3390/jcm15176878

**Published:** 2026-09-05

**Authors:** Colin McQuade, Dayana Davoudi, Katherine Lajkosz, Laura Donahoe, John Granton, Marc de Perrot, Micheal McInnis

**Affiliations:** 1 Division of Cardiothoracic Imaging, Department of Medical Imaging, University of Toronto, Toronto, ON M5S 1A1, Canada; 2 Body Imaging Division, Department of Radiology, Beaumont Hospital, D09 Dublin, Ireland; 3 Faculty of Medicine, University of Toronto, Toronto, ON M5S 1A1, Canada; 4 Biostatistics Research Unit, University Health Network, Toronto, ON M5G 2C4, Canada; 5 Division of Thoracic Surgery, Department of Surgery, University of Toronto, Toronto, ON M5S 1A1, Canada; 6 Division of Respirology, Department of Medicine, University of Toronto, Toronto, ON M5S 1A1, Canada; 7 University Medical Imaging Toronto, Toronto General Hospital, Toronto, ON M5G 2C4, Canada

**Keywords:** chronic thromboembolic pulmonary disease, chronic thromboembolic pulmonary hypertension, CT pulmonary angiography, pulmonary embolism, vascular obstruction

## Abstract

**Background**: Chronic thromboembolic disease is a recognised sequela of acute pulmonary embolism and may occur with or without pulmonary hypertension. The purpose of this study was to evaluate differences in chronic thromboembolic burden, lesion type, and distribution between chronic thromboembolic pulmonary disease without pulmonary hypertension (CTEPD) and chronic thromboembolic pulmonary hypertension (CTEPH). **Methods**: This retrospective single-centre study included patients with CTEPD or CTEPH by right heart catheterisation using 2022 ESC/ERS criteria between 2021 and 2022. Two CTEPH patients were randomly selected per CTEPD patient. CTPA studies were reviewed by a blinded thoracic radiologist, assessing 32 pulmonary vessels from the main to segmental arteries for chronic thromboembolic lesions. Groups were compared by disease distribution, most proximal lesion level, Qanadli obstruction index, lesion type, and location. Exploratory receiver operating characteristic (ROC) analysis assessed discriminatory performance. **Results**: The 44 CTEPD and 88 CTEPH patients included had no differences in age, BMI, or sex. CTEPH patients had shorter six-minute walk distance (*p* = 0.001), greater right ventricular dilation/dysfunction (*p* < 0.001), and lower prevalence of deep vein thrombosis (*p* = 0.03). CTPA identified more lesions in CTEPH (21.2 vs. 10.0 lesions/case, *p* < 0.001) across more lobes (4.8 vs. 3.4, *p* < 0.001), with more proximal main/lobar disease (*p* < 0.001). CT obstruction index was 51% in CTEPH and 26% in CTEPD (*p* < 0.001). Total lesion number best discriminated CTEPH from CTEPD (AUC 0.91; optimal cutoff, 16 lesions). **Conclusions**: CTEPH demonstrates a greater, more diffuse, and more proximal chronic thromboembolic lesion burden on CTPA than CTEPD.

## 1. Introduction

Chronic thromboembolic pulmonary hypertension (CTEPH) is a complication known to occur in approximately 3% of patients who survive an episode of acute pulmonary embolism [1,2]. The disorder is characterised by chronic pulmonary embolism (PE) on imaging, despite adequate anticoagulation in patients meeting clinically defined haemodynamic criteria. In 2022, the mean pulmonary artery pressure threshold for pulmonary hypertension was revised from ≥25 mmHg to >20 mmHg by the European Society of Cardiology and European Respiratory Society (ESC/ERS) [3]. As a result, an increased number of acute PE survivors may now be classified as having CTEPH rather than chronic thromboembolic pulmonary disease without pulmonary hypertension (CTEPD). Within the spectrum of chronic thromboembolic disease, patients may have pulmonary hypertension at rest, termed CTEPH, or remain without resting pulmonary hypertension, herein referred to as chronic thromboembolic pulmonary disease (CTEPD).

Patients with CTEPH may present with a gradual progression of symptoms, including dyspnoea on exertion and decreased exercise tolerance, which is related to impaired cardiac output as well as increased dead space ventilation [4]. However, a lack of awareness among patients and clinicians, including radiologists, often leads to under-recognition of CTEPH and, therefore, delays in diagnosis. Delays in CTEPH diagnosis have been shown to correlate with both shorter survival time from diagnosis as well as less favourable haemodynamic profiles [5]. Although pulmonary hypertension requires invasive confirmation by right heart catheterisation, CT pulmonary angiography (CTPA) provides a non-invasive assessment of chronic thromboembolic lesion burden, morphology, and distribution. Identifying CTEPH-associated imaging features could raise diagnostic suspicion and prompt appropriate haemodynamic and multidisciplinary evaluation.

Prior studies comparing CTPA findings in CTEPD and CTEPH have reported inconsistent differences in chronic thromboembolic burden and distribution [6,7,8,9]. Although differences in selected imaging and perfusion features have been described, the extent to which the number, morphology, and anatomic distribution of individual chronic thromboembolic lesions differ between CTEPD and CTEPH remains incompletely understood. For example, Capone et al. found no difference in vascular obstruction burden but only used a modified Qanadli index which classifies lesions as obstructive of non-obstructive and is a composite score where information on lesion location and type is lost [7]. Indeed, in clinical practice, it is observed that some patients with CTEPD may have a large clot burden, whereas some patients with CTEPH have a relatively low clot burden on CTPA. An understanding of differences in clot burden may help clarify the pathogenesis of CTEPH and assist radiologists in distinguishing CTEPH from CTEPD.

## 2. Materials and Methods

### 2.1. Study Design and Population

Institutional Research Ethics Board approval was obtained (REB# 23-5614), the requirement for informed consent was waived, and the study was conducted in accordance with the Declaration of Helsinki and applicable institutional regulations. This was a retrospective, single-centre study performed at a quaternary referral centre for chronic thromboembolic disease. Adult patients evaluated by the institutional multidisciplinary chronic thromboembolic pulmonary hypertension team between 1 January 2021 and 31 December 2022 were identified from the electronic medical record based on their multidisciplinary diagnosis. All eligible patients with chronic thromboembolic pulmonary disease without pulmonary hypertension (CTEPD) were included. For each patient with CTEPD, two eligible patients with chronic thromboembolic pulmonary hypertension (CTEPH) were selected by random number generation in Excel (2016, Microsoft, Redmond, WA, USA) from the eligible CTEPH cohort. There were more patients with a diagnosis of CTEPH, allowing 2:1 sampling to improve statistical power.

### 2.2. Definitions of CTEPD and CTEPH

The multidisciplinary team comprised thoracic radiologists, thoracic surgeons, respirologists, and allied healthcare professionals with expertise in CTEPH. The diagnosis of chronic thromboembolic disease required symptoms attributable to chronic thromboembolic disease and imaging evidence of chronic organised pulmonary arterial obstruction after at least 3 months of therapeutic anticoagulation [3].

The reference standard was multidisciplinary classification informed by resting right heart catheterisation (RHC) and chronic thromboembolic imaging findings using the 2022 European Society of Cardiology/European Respiratory Society haemodynamic framework [3]. Thus, the diagnosis of pulmonary hypertension required a mean pulmonary artery pressure of >20 mmHg, a pulmonary capillary wedge pressure ≤ 15 mmHg and a pulmonary vascular resistance > 2 WU. Patients with chronic thromboembolic disease and resting pulmonary hypertension were classified as CTEPH, whereas those without resting pulmonary hypertension were classified as CTEPD. RHC-derived mean pulmonary arterial pressure, pulmonary vascular resistance, and pulmonary capillary wedge pressure are reported in Table 1.

### 2.3. Patient Selection and Data Collection

Patients were excluded if (1) RHC and CT pulmonary angiography (CTPA) were not performed within 6 months of each other; (2) CTPA quality was inadequate because of main pulmonary artery attenuation < 200 HU or motion artefact obscuring segmental vessels; (3) symptoms were attributable to a condition other than chronic thromboembolic disease; or (4) pulmonary endarterectomy or balloon pulmonary angioplasty had been performed before the evaluated CTPA and RHC.

Clinical data were retrieved from the electronic medical record and included baseline demographics, 6 min walk distance, resting RHC haemodynamic parameters, echocardiographic findings, B-type natriuretic peptide concentration, and comorbidities. Echocardiographic right ventricular dilation and systolic dysfunction were classified according to the assessment documented by the interpreting cardiologist in the final echocardiography report. Values closest to the initial multidisciplinary consultation were used.

### 2.4. CTPA Acquisition and Interpretation

CTPA was the index test evaluated in this study. For examinations performed at our institution, imaging was acquired during suspended respiration after intravenous administration of 70 mL of iodinated contrast material, Ultravist 370 (Bayer Healthcare, Leverkusen, Germany), at 5 mL/s through an intravenous cannula of 18 gauge. Bolus tracking was performed with a region of interest placed in the main pulmonary artery, and image acquisition was automatically initiated when pulmonary arterial attenuation reached ≥200 HUs. The standard tube voltage was 120 kV, although contrast volume and tube voltage were adjusted when required according to patient-specific factors, including body weight. Automated exposure control was used to optimise radiation dose. Images were reconstructed with a mediastinal kernel at a transverse section thickness of 0.5–1.0 mm and coronal and sagittal section thickness of 1.0 mm.

As a quaternary referral centre for the surgical and percutaneous management of chronic thromboembolic disease, some patients underwent CTPA at external institutions, resulting in technical variation among imaging protocols. External examinations were included only when they met the predefined minimum image-quality requirements, including adequate pulmonary arterial opacification, absence of motion artefact obscuring segmental vessels, and a reconstructed section thickness of 1.5 mm or less. If the study did not meet predefined minimum image-quality criteria that might influence lesion detection, then the study was excluded.

### 2.5. Lesion Classification

Following initial screening of CTPA studies for technical quality by a thoracic radiology fellow (CM), all examinations were evaluated by an expert thoracic radiologist (MM) with experience in CTEPH who was blinded to the multidisciplinary haemodynamic classification. The pulmonary arterial tree was analysed as 32 distinct named vessels to the level of the segmental pulmonary arteries in each patient, in accordance with a previously described approach [10]. The 32-vessel model comprised three main, five lobar, one interlobar, two basal trunks, one lingular, and twenty segmental pulmonary arteries. Each named vessel was assessed for chronic thromboembolic lesions, classified as complete occlusion, eccentric wall thickening, or web/slit. In cases where there was more than one lesion type present per vessel, only the most occlusive lesion was recorded. For pulmonary segments without an identifiable segmental lesion, the subsegmental arteries were subsequently assessed. Because of their smaller calibre, subsegmental disease was recorded as present or absent rather than classified by lesion morphology.

### 2.6. CTPA Scoring

Lesion type and location informed calculation of two separate pulmonary arterial tree thromboembolic scoring systems: the CT obstruction index (CTOI) and the CT level of disease (CTLD). The CTOI, a modified version of the Qanadli index, denotes the burden of vascular obstruction [11,12]. The CTLD is the radiologic surrogate for the University of California, San Diego (UCSD) classification system for CTEPH. In this, only the single most proximal lesion denoted the level of disease [10,13,14]. CTLD level 1 indicated main pulmonary artery disease; level 2, lobar or interlobar disease; level 3, segmental-level disease; and level 4, disease confined to the subsegmental pulmonary arteries.

### 2.7. Statistical Analysis

Continuous variables were summarised as mean ± standard deviation and assessed for normality before between-group comparison. Normally distributed continuous variables were compared using the independent-samples Student *t* test, and non-normally distributed variables were compared using the Mann–Whitney U test. Categorical variables were compared using the chi-square test. Individuals with missing data points were excluded from those particular statistical tests.

Receiver operating characteristic analysis was performed as an exploratory analysis to assess the discriminatory performance of radiologic imaging features between CTEPH and CTEPD. For each imaging feature, the area under the receiver operating characteristic curve was calculated together with a data-derived optimal cut point. The optimal cut point was derived using Youden’s Index. Logistic regression models were adjusted for age, sex, and history of DVT. Two-sided *p* < 0.05 was considered statistically significant. Statistical analyses were performed using R version 4.2.2.

## 3. Results

### 3.1. Baseline Clinical Variables

**Figure 1:**
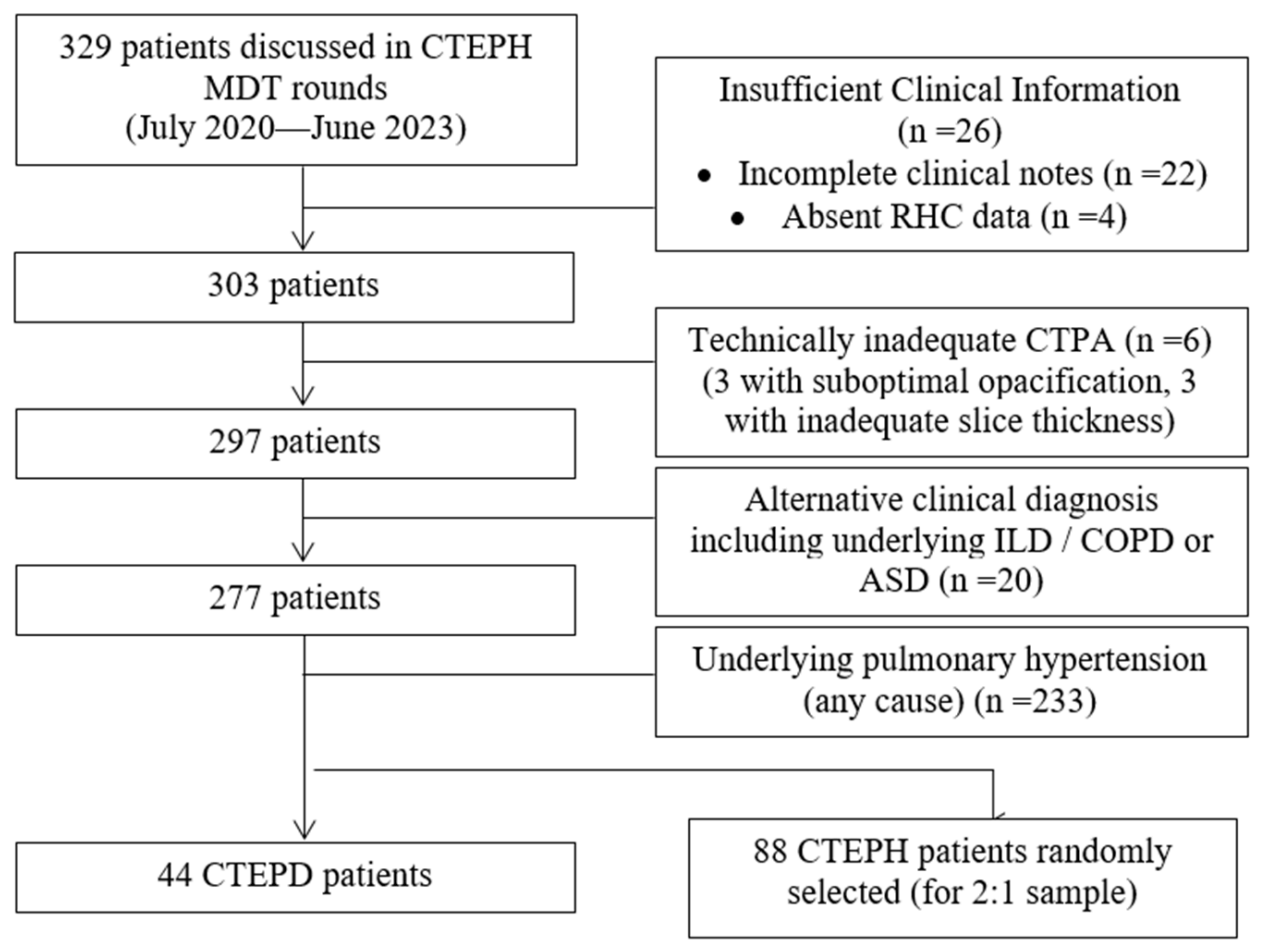
Flow diagram of patient selection and study cohort formation. The diagram shows patient identification, eligibility assessment, exclusions, and final inclusion of 44 patients with chronic thromboembolic pulmonary disease without pulmonary hypertension and 88 patients with chronic thromboembolic pulmonary hypertension.

After application of the eligibility criteria, 132 patients were included in the analysis, comprising 44 patients with CTEPD and 88 with CTEPH (Figure 1). Half were male (n = 66, 50%), and the groups were similar in age, with a mean age of 55.7 ± 16.0 years in patients with CTEPD and 58.7 ± 15.4 years in those with CTEPH (*p* = 0.38). Age, sex, and body mass index did not significantly differ between groups (Table 1). Most patients had New York Heart Association functional class II or III symptoms. Patients with CTEPH had a shorter 6 min walk distance than those with CTEPD (379.4 ± 122.5 vs. 463.3 ± 126.2 m, *p* = 0.001). A history of deep vein thrombosis was more frequent in CTEPD (52% vs. 33%, *p* = 0.03), with no other significant differences in recorded comorbidities (Table S1).

On echocardiography, right ventricular dilation and systolic dysfunction, as classified in the finalised echocardiography report by the interpreting cardiologist were more common in patients with CTEPH (both *p* < 0.001), although a minority of patients with CTEPD had right ventricular dilation (n = 9, 21%) or dysfunction (n = 4, 9%). Time from CT to RHC was similar between groups (2 [1,3] months vs. 2 [1,3] months, *p* = 0.70). Resting RHC demonstrated no pulmonary hypertension in patients with CTEPD and confirmed pulmonary hypertension in all patients with CTEPH (mPAP 41.5 ± 10.8 mmHg). Mean B-type natriuretic peptide was higher in CTEPH than CTEPD (266.7 vs. 43.7 pg/mL, *p* < 0.001) (Table 1).

### 3.2. Chronic Pulmonary Embolism Type and Distribution

Chronic pulmonary emboli were common in all lobes but most commonly found in the right lower lobe in both CTEPH patients (99%) and CTEPD patients (93%) (Table 2). The right middle lobe was the least commonly involved lobe in patients with CTEPD (36%), although it was involved in 94% of CTEPH patients. Overall, CTEPH was a more diffuse process with chronic PE typically found in all lobes compared to patients with CTEPD (4.8 ± 0.6 vs. 3.4 ± 1.3 lobes/case, *p* < 0.001) (Table 2).

The CTLD differed between CTEPD and CTEPH (Table 2). Most patients with CTEPD (61%) were CTLD 3 with the most proximal disease in the segmental vessels. Among CTEPH patients, most were CTLD 2 (53%) and 17% had CTLD 1. CTLD 4, a disease only found in the subsegmental vasculature, was rare in both CTEPH and CTEPD.

Among 4224 named main-to-segmental pulmonary vessels, 1805 chronic thromboembolic lesions were identified. An additional 501 subsegmental lesions were detected in pulmonary segments without identifiable segmental disease, yielding 2306 lesions overall. The CT obstruction index was nearly twofold higher in CTEPH than in CTEPD (51% vs. 26%), corresponding to approximately twice the vascular obstruction. On average, each case of CTEPH had twice as many identifiable lesions as CTEPD in the 32 possible pulmonary vessels and extending to the subsegmental level (21.2 ± 5.6 vs. 10.0 ± 5.6, *p* < 0.001). Webs, eccentric wall thickening, occlusions and subsegmental lesions were all more common in CTEPH (Table 2). Webs made up a greater proportion of the lesions in CTEPD compared to CTEPH (50.5 vs. 43.2%, *p* = 0.007). Occlusions were the least common type of lesion among CTEPD patients and the proportion of occlusive lesions was lower in CTEPD compared to CTEPH (11.4 vs. 18.5%, *p* < 0.001) (Table 3).

### 3.3. Receiver Operating Characteristic Curve Analysis

**Figure 2:**
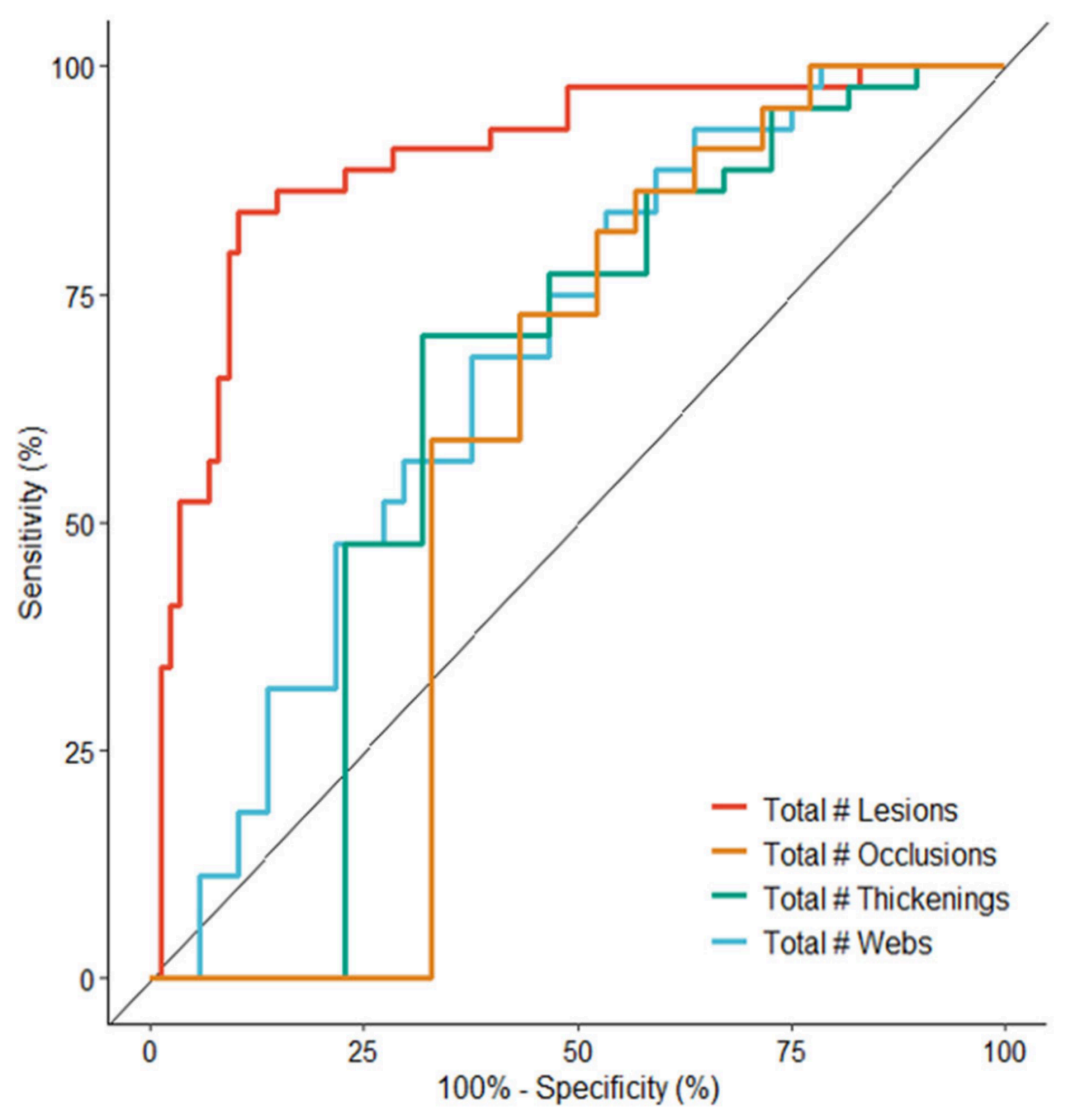
Receiver operating characteristic analysis of chronic thromboembolic lesion burden on CT pulmonary angiography. Curves show total lesion count and counts of occlusions, eccentric wall thickening, and webs for discrimination between chronic thromboembolic pulmonary disease without pulmonary hypertension and chronic thromboembolic pulmonary hypertension. The diagonal line indicates no discrimination.

In the segmental vessels, there were, again, more lesions of all types in CTEPH compared to CTEPD. Among pulmonary segments without identifiable segmental disease, subsegmental lesions were present in 394 of 763 segments (51.6%) in CTEPH and 107 of 709 segments (15.1%) in CTEPD (Table 4). Exploratory ROC analysis showed that total lesion count had the highest observed discrimination between CTEPH and CTEPD (AUC, 0.91, 95%CI 0.85–0.96). The data-derived threshold of 16 lesions yielded 90% sensitivity and 84% specificity (Figure 2). The diagnostic performance persists when adjusting for age, sex, and history of DVT (AUC 0.93, 95%CI 0.88–0.98).

## 4. Discussion

This study identified CTEPH and CTEPD as radiologically distinct entities, with twice as much vascular obstruction seen in CTEPH compared to CTEPD on CTPA. Patients with CTEPH have more proximal disease than CTEPD with most patients having disease at the main and lobar level, UCSD level 1 and 2, compared to most patients with CTEPD having disease at the segmental level, UCSD level 3. Patients with CTEPH were more likely to have occlusive disease, while non-occlusive webs contributed to a greater proportion of the lesions in CTEPD. CTEPH was also a more diffuse disease than CTEPD and the contrast between CTEPH and CTEPD was most striking at the subsegmental level, where most of the pulmonary segments evaluated in CTEPH had subsegmental lesions. Total lesion count showed strong observed discrimination between groups, supporting an association between greater macroscopic thromboembolic burden and the CTEPH phenotype.

Other groups have previously identified radiologic differences between patients with CTEPH and CTEPD. Saeedan et al. found more perfusion defects on dual-energy CT among patients with CTEPH, although vascular obstruction did not significantly differ between groups; unilateral disease was more common in CTEPD [6]. Capone et al. similarly found no significant difference in vascular obstruction, although mosaic attenuation was more common in CTEPH [7]. A prospective 2019 study including 36 patients with CTEPD found a significantly lower CT obstruction index in CTEPD than CTEPH but did not further characterise lesion morphology or distribution [8]. Differences from prior studies may reflect variation in cohort selection, haemodynamic definitions, and the granularity of CTPA analysis. Our vessel-by-vessel assessment quantified individual lesion morphology and distribution throughout the pulmonary arterial tree; however, the relative contribution of these methodological and population differences remains uncertain. More recently, Cerrone et al. found no significant differences in chronic thromboembolic burden or distribution across CTEPD haemodynamic phenotypes defined using contemporary thresholds, further highlighting heterogeneity within the CTEPD spectrum [9].

Detailed characterisation of chronic thromboembolic lesion burden, morphology, and distribution in CTEPD is increasingly important as emerging evidence suggests that carefully selected symptomatic patients may benefit from balloon pulmonary angioplasty (BPA) or pulmonary endarterectomy (PEA). Small BPA cohorts have demonstrated improvements in pulmonary vascular haemodynamics and functional status despite near-normal resting pulmonary artery pressures at baseline. In a 2018 study of 10 patients with a mean baseline mPAP of 21 mmHg, BPA significantly reduced PVR (*p* = 0.004) and improved functional class, although mPAP did not change significantly [15]. Similarly, among 15 patients with a median baseline mPAP of 20 mmHg, BPA was associated with improvements in mPAP (*p* = 0.001), PVR (*p* = 0.04), functional class (*p* < 0.001), and 6 min walk distance (*p* = 0.03), although long-term survival was not assessed [16]. The literature evaluating PEA in CTEPD remains limited but has shown similar haemodynamic and functional benefits. In a cohort of 42 patients, median mPAP decreased from 21 to 18 mmHg after PEA (*p* < 0.05), accompanied by significant improvements in PVR, functional class, and 6 min walk distance (all *p* < 0.05) [17]. Comparable findings have been reported in other cohorts. Among 48 patients with a mean preoperative mPAP of 20 mmHg, PEA significantly improved mPAP (*p* = 0.002), PVR (*p* < 0.001), functional status (*p* < 0.001), and 6 min walk distance at 1 year [18]. A separate study of 23 patients with a mean baseline mPAP of 21 mmHg likewise demonstrated significant postoperative improvements in mPAP, PVR, functional status, and 6 min walk distance (all *p* < 0.001) [19]. Collectively, these studies suggest that anatomically targeted intervention may improve haemodynamic and functional outcomes in selected patients with CTEPD.

Radiologists should recognise the potential clinical importance of chronic PE even in the absence of pulmonary hypertension. In our cohort, NYHA functional class was comparable between patients with CTEPD and CTEPH, indicating substantial functional limitation in both groups. The availability of RHC in all patients with CTEPD strengthened haemodynamic classification; however, because RHC is more likely to be performed in symptomatic or functionally impaired patients referred for specialist assessment, our CTEPD cohort may represent a more severely affected subset of the broader CTEPD population, limiting generalisability. Radiologists should therefore recommend clinical evaluation in symptomatic patients with chronic PE. At our institution, this includes multidisciplinary assessment by the CTEPH team.

We used CTPA to quantify chronic PE extent and distribution as it has been used in several prior studies and is a reliable indicator of UCSD surgical level of disease [14]. Accurately identifying the level of disease before intervention can assist with operative planning, inform the likelihood of surgical success, and identify those patients who may need ongoing targeted medical therapy for the management of residual pulmonary hypertension postoperatively. CT may also help to identify those patients for whom PEA may not be as beneficial and for whom balloon pulmonary angioplasty or medical therapy may be preferred.

Differences between CTEPH and CTEPD were not limited only to the number and proximity of chronic PE. We found a greater proportion of total occlusions in CTEPH, whereas CTEPD lesions were predominantly web-like, which are presumably not as impactful on the pulmonary vascular flow. We also found that CTEPH lesions in the main and lobar vessels were more commonly characterised as eccentric thickening rather than web-like or occlusive disease. Although wall thickening may be obstructive to flow, vessel wall stiffening caused by organised thrombus along the walls of proximal vessels likely contributes to pulmonary hypertension. Finally, subsegmental lesions were substantially more common in CTEPH than CTEPD, suggesting that distal thromboembolic burden is an important component of the CTEPH imaging phenotype. While we have outlined key differences between CTEPH and CTEPD, much work is required in external validation. Systematic lesion analysis would potentially complement RHC assessment in phenotyping patients and future studies could evaluate the impact of this systematic analysis on treatment and outcome.

Because we used conventional CTPA, we were unable to evaluate microvascular lesions in the lung, which are crucial in the pathogenesis of CTEPH and not captured by our study. Using a photon-counting detector CT, Remy-Jardin et al. describe high-resolution CT findings in a cohort of 29 individuals with CTEPH and suggest that small-vessel changes detectable by this new CT technology [20]. A future focus of research thus should be comparing CTEPH to CTEPD using photon-counting detector CT or other new cardiovascular imaging approaches, including the use of artificial intelligence [21]. This would also include evaluating imaging at presentation with acute PE as we observed a high prevalence of deep vein thrombosis in the cohort with CTEPD compared to CTEPH (52% vs. 33%, *p* = 0.03) and initial thromboembolism phenotype may thus play a role in the subsequent hemodynamic phenotype.

Limitations of our study include the retrospective nature of the study design performed at a single, albeit quaternary referral, centre. The CTEPD patients in our study are essentially a subset of the broader population of CTEPD patients. The fact that they were assessed at the CTEPH clinic in our institution implies they are more symptomatic than a broader selection of patients with CTEPD and further study is required to determine if similar findings would be present in an unselected population. Patients with exercise-induced CTEPH were not separately assessed but are an important cohort for future evaluation. CTPA interpretation was also performed by a single expert thoracic radiologist and inclusion of external CTs could create technical variations. Although a single reader ensured consistency and blinding to haemodynamic classification, lesion characterisation, particularly of webs and distal disease, may be subject to interobserver variability. External validation of the findings is required.

## 5. Conclusions

In conclusion, CTEPH was associated with greater, more diffuse, and more proximal chronic thromboembolic lesion burden on CT than CTEPD. Total lesion count showed strong observed discrimination between groups but requires external validation before use as a diagnostic threshold. Detailed CT assessment of lesion burden and distribution provides objective imaging information relevant to haemodynamic and multidisciplinary evaluation.

## Figures and Tables

**Table 1:** Baseline clinical, haemodynamic, and echocardiographic characteristics of patients with CTEPD and CTEPH.

	CTEPD (n = 44)	CTEPH (n = 88)	*p*-Value
Sex			0.85
Male	21 (47.7)	45 (51.1)	
Female	23 (52.3)	43 (48.9)	
Age (years)	55.7 ± 16.0	58.7 ± 15.4	0.38
BMI ^a^	29.9 ± 7.4	29.7 ± 6.5	0.94
NYHA Class ^b^			0.73
I	3 (9.4)	5 (6.6)	
II	14 (43.8)	29 (38.2)	
III	14 (43.8)	35 (46.1)	
IV	1 (3.1)	7 (9.2)	
6MWT (m) ^c^	463.3 ± 126.2	379.4 ± 122.5	0.001
BNP (pg/mL) ^d^	43.7 ± 71.5	266.7 ± 383.0	<0.001
Right Heart Catheterisation			
mPAP (mmHg)	15.3 ± 3.8	41.5 ± 10.8	<0.001
PVR (Wood units) ^e^	1.5 ± 0.8	6.0 ± 3.0	<0.001
PCWP (mmHg) ^f^	7.6 ± 3.8	11.8 ± 8.9	<0.001
Echocardiogram			
RV dilation ^g^	9 (21.4)	59 (72.8)	<0.001
RV dysfunction ^h^	4 (9.3)	50 (61.7)	<0.001
RVSP (mmHg) ^i^	31.5 ± 8.2	68.4 ± 21.8	<0.001

**Note:** Data are presented as mean ± standard deviation or n (%), unless otherwise indicated. Percentages were calculated among patients with available data for each variable. BMI, body mass index; BNP, B-type natriuretic peptide; CTEPD, chronic thromboembolic pulmonary disease; CTEPH, chronic thromboembolic pulmonary hypertension; 6MWD, 6 min walk distance; mPAP, mean pulmonary arterial pressure; NYHA, New York Heart Association; PCWP, pulmonary capillary wedge pressure; PVR, pulmonary vascular resistance; RV, right ventricular; RVSP, right ventricular systolic pressure. Missing data were as follows: ^a^ n = 6 (CTEPD, n = 0; CTEPH, n = 6); ^b^ n = 24 (CTEPD, n = 12; CTEPH, n = 12); ^c^ n = 17 (CTEPD, n = 5; CTEPH, n = 12); ^d^ n = 43 (CTEPD, n = 15; CTEPH, n = 28); ^e^ n = 56 (CTEPD, n = 18; CTEPH, n = 38); ^f^ n = 7 (CTEPD, n = 2; CTEPH, n = 5); ^g^ n = 9 (CTEPD, n = 2; CTEPH, n = 7); ^h^ n = 8 (CTEPD, n = 1; CTEPH, n = 7); and ^i^ n = 45 (CTEPD, n = 22; CTEPH, n = 23).

**Table 2:** CTPA distribution, CT level of disease, and chronic thromboembolic lesion burden in CTEPD and CTEPH.

Characteristic	CTEPD (n = 44)	CTEPH (n = 88)	*p*-Value
**Disease Distribution**			
Involved lobes, n	3.4 ± 1.3	4.8 ± 0.6	<0.001
Right upper lobe	27 (61.4)	85 (96.6)	<0.001
Right middle lobe	16 (36.4)	83 (94.3)	<0.001
Right lower lobe	41 (93.2)	87 (98.9)	0.11
Left upper lobe	31 (70.5)	84 (95.5)	<0.001
Left lower lobe	35 (79.5)	86 (97.7)	0.001
**CT Level of Disease**			<0.001
Level 1	1 (2.3)	15 (17.0)	
Level 2	15 (34.1)	47 (53.4)	
Level 3	27 (61.4)	24 (27.3)	
Level 4	1 (2.3)	2 (2.3)	
**Mean Number of Lesions per Case**			
Number of lesions	10.0 ± 5.6	21.2 ± 5.6	<0.001
Number of webs	5.0 ± 4.0	9.2 ± 5.9	<0.001
Number of eccentric thickening	1.4 ± 2.0	3.6 ± 3.5	<0.001
Number of occlusions	1.1 ± 1.8	3.9 ± 4.6	<0.001
Number of subsegmental	2.4 ± 2.0	4.5 ± 3.6	0.002

**Note:** Data are presented as mean ± standard deviation or n (%). The CT level of disease represents the most proximal chronic thromboembolic lesion identified on CTPA and was compared between groups as an overall categorical variable. Level 1 indicates main pulmonary artery disease; Level 2, lobar or interlobar disease; Level 3, segmental disease; and Level 4, disease confined to the subsegmental pulmonary arteries. CTEPD = chronic thromboembolic pulmonary disease; CTEPH = chronic thromboembolic pulmonary hypertension; CTPA = computed tomography pulmonary angiography.

**Table 3:** Distribution of chronic thromboembolic lesion types in CTEPD and CTEPH.

	Full Sample (n = 2306 Lesions)	CTEPD (n = 440 Lesions)	CTEPH (n = 1866 Lesions)	*p*-Value
**Lesion Type**				<0.001
Web/slit	1029 (44.6)	222 (50.5)	807 (43.2)	0.007
Eccentric thickening	381 (16.5)	61 (13.9)	320 (17.1)	0.11
Occlusion	395 (17.1)	50 (11.4)	345 (18.5)	<0.001
Subsegmental	501 (21.7)	107 (24.3)	394 (21.1)	0.16

**Note:** Data are presented as n (%), with percentages calculated by column and representing the distribution of lesion types within each group.

**Table 4:** Anatomic distribution and chronic thromboembolic lesion types in CTEPD and CTEPH.

	Full Sample	CTEPD	CTEPH	*p*-Value
**Main Pulmonary Arteries**	n = 396 vessels	n = 132 vessels	n = 264 vessels	<0.001
Web	1 (0.3)	1 (0.8)	0 (0.0)	
Eccentric Thickening	23 (5.8)	0 (0.0)	23 (8.7)	
Occlusion	3 (0.8)	0 (0.0)	3 (1.1)	
No Lesion	369 (93.2)	131 (99.2)	238 (90.2)	
**Lobar/Interlobar Arteries**	n = 792 vessels	n = 264 vessels	n = 528 vessels	<0.001
Web	37 (4.7)	14 (5.3)	23 (4.4)	
Eccentric Thickening	144 (18.2)	13 (4.9)	131 (24.8)	
Occlusion	33 (4.2)	2 (0.8)	31 (5.9)	
No Lesion	578 (73.0)	235 (89.0)	343 (65.0)	
**Segmental Arteries**	n = 3036 vessels	n = 1012 vessels	n = 2024 vessels	<0.001
Web	991 (32.6)	207 (20.5)	784 (38.7)	
Eccentric Thickening	214 (7.0)	48 (4.7)	166 (8.2)	
Occlusion	359 (11.8)	48 (4.7)	311 (15.4)	
No Lesion	1472 (48.5)	709 (70.1)	763 (37.7)	
**Subsegmental-level Arteries**	n = 1472 segments	n = 709 segments	n = 763 segments	<0.001
Subsegmental	501 (34.0)	107 (15.1)	394 (51.6)	
No Lesion	971 (66.0)	602 (84.9)	369 (48.4)	

**Note:** Data are presented as n (%), with percentages calculated within each anatomic level and study group. *p* values represent comparisons of the overall distribution of lesion categories between CTEPD and CTEPH at each anatomic level. Segmental-level arteries included the 20 segmental pulmonary arteries, two basal trunks, and one lingular artery assessed per patient. Subsegmental arteries were assessed only in pulmonary segments without an identifiable segmental lesion. CTEPD, chronic thromboembolic pulmonary disease; CTEPH, chronic thromboembolic pulmonary hypertension.

## Data Availability

Data may be available upon request from the corresponding author.

## Supplementary Materials

The following supporting information can be downloaded at: https://www.mdpi.com/article/10.3390/jcm15176878/s1, Table S1: comorbidities and clinical risk factors in patients with CTEPD and CTEPH.

## Abbreviations

The following abbreviations are used in this manuscript:

6MWD	Six-minute walk distance
BNP	B-type natriuretic peptide
CTED	Chronic thromboembolic disease
CTEPD	Chronic thromboembolic pulmonary disease
CTEPH	Chronic thromboembolic pulmonary hypertension
CTLD	Computed tomography level of disease
CTOI	Computed tomography obstruction index
CTPA	Computed tomography pulmonary angiography
ESC/ERS	European Society of Cardiology/European Respiratory Society
mPAP	Mean pulmonary arterial pressure
NYHA	New York Heart Association
PCWP	Pulmonary capillary wedge pressure
PE	Pulmonary embolism
PH	Pulmonary hypertension
PVR	Pulmonary vascular resistance
RHC	Right heart catheterisation
ROC	Receiver operating characteristic
RV	Right ventricle/right ventricular
RVSP	Right ventricular systolic pressure
UCSD	University of California, San Diego
