# Supplementary material for: Differences in Chronic Pulmonary Embolism Burden in Chronic Thromboembolic Disease with and Without Pulmonary Hypertension
Source: J Clin Med. 2026 Sep 5;15(17):6878. doi: 10.3390/jcm15176878 (PMC13566429; doi:10.3390/jcm15176878)
Supplement: Supplementary file 1 [file jcm-15-06878-s001.zip › jcm-4523172-supplementary.pdf]

Supplementary Table S1. Comorbidities and Clinical Risk Factors in Patients With CTEPD and CTEPH

**Comorbidities**	**CTEPD (n =44)**	**CTEPH (n =88)**	**p-value**
Deep venous thrombosis	23 (52)	29 (33)	0.03
Antiphospholipid syndrome	3 (6.8)	5 (5.7)	1
Sickle cell disease	0 (0)	2 (2.3)	0.55
Thalassemia	1 (2.3)	1 (1.1)	1
Lupus anticoagulant	0 (0)	1 (1.1)	1
Factor V Leiden	1 (2.3)	3 (3.4)	1
Antithrombin III deficiency	1 (2.3)	1 (1.1)	1
Splenectomy	0 (0)	2 (2.3)	0.55
Inflammatory bowel disease	1 (2.3)	3 (3.4)	1
Lymphoproliferative	1 (2.3)	1 (1.1)	1
Infective endocarditis	0 (0)	2 (2.3)	0.55
Malignancy	2 (4.5)	6 (6.8)	0.72
Lung disease	2 (4.5)	7 (8.0)	0.72
Diabetes mellitus	1 (2.3)	9 (10)	0.16
Cardiovascular disease	2 (4.5)	6 (6.8)	0.72
Hypertension	5 (11)	15 (17)	0.39
TIA / CVA	0 (0)	4 (4.5)	0.30
Arrhythmia	0 (0)	3 (3.4)	0.55
Thyroid disease	4 (9.1)	11 (13)	0.56
PFO / ASD	0 (0)	3 (3.4)	0.55

Note: Data are presented as n (%), with percentages calculated by column. Between-group comparisons were performed using categorical statistical tests as appropriate. CTEPD, chronic thromboembolic pulmonary disease; CTEPH, chronic thromboembolic pulmonary hypertension; CVC, central venous catheter; PFO, patent foramen ovale; ASD, atrial septal defect; TIA, transient ischemic attack; CVA, cerebrovascular accident. N/A indicates that a between-group comparison was not applicable because no events occurred in either group.
